# Tumour-Secreted Hsp90α on External Surface of Exosomes Mediates Tumour - Stromal Cell Communication via Autocrine and Paracrine Mechanisms

**DOI:** 10.1038/s41598-019-51704-w

**Published:** 2019-10-22

**Authors:** Xin Tang, Cheng Chang, Jiacong Guo, Vadim Lincoln, Chengyu Liang, Mei Chen, David T. Woodley, Wei Li

**Affiliations:** 1 Department of Dermatology and the USC-Norris Comprehensive Cancer Center, University of Southern California Keck Medical Centre, Los Angeles, CA 90033 USA; 2 Department of Molecular Microbiology & Immunology, the USC-Norris Comprehensive Cancer Centre, University of Southern California Keck Medical Centre, Los Angeles, CA 90033 USA

**Keywords:** Breast cancer, Diagnostic markers

## Abstract

Extracellular heat shock protein-90alpha (eHsp90α) plays an essential role in tumour invasion and metastasis. The plasma eHsp90α levels in patients with various cancers correlate with the stages of the diseases. Nonetheless, the mechanism of action by tumour-secreted eHsp90α remained unclear. Here we show that eHsp90α accounts for approximately 1% of the total cellular Hsp90α and is associated with tumour-secreted exosomes. CRISPR-cas9 knockout of Hsp90α did not affect the overall distribution and quantity of secreted exosomes, but it caused increased exosome-associated CD9 and decreased exosome-associated TSG101, Alix, and CD63. However, Hsp90α-knockout tumour cells have not only lost their own constitutive motility, but also the ability to recruit stromal cells via secreted exosomes. These defects are specifically due to the lack of eHsp90α on tumour cell-secreted exosomes. Anti-Hsp90α antibody nullified the pro-motility activity of tumour-secreted exosomes and human recombinant Hsp90α protein fully rescued the functional defects of eHsp90α-free exosomes. Finally, while current exosome biogenesis models exclusively implicate the luminal location of host cytosolic proteins inside secreted exosomes, we provide evidence for eHsp90α location on the external surface of tumour-secreted exosomes. Taken together, this study elucidates a new mechanism of action by exosome-associated eHsp90α.

## Introduction

Mammals have two genes that encode the two cytosolic heat shock protein (Hsp90) proteins, Hsp90α and Hsp90β, Together, these two proteins account for 2–3% of the total amount of cellular proteins in normal cells and up to 7% in tumour cells^1,2^ (http://www.picard.ch). Germline Hsp90β gene knockout causes defects in the placental labyrinth and subsequent embryonic death in mice^3^. In contrast, mice with reported Hsp90α knockout are phenotypically normal, albeit certain tissue-specific defects^4,5^. In tumour cells, CRISPR/Case9 knockout of Hsp90β resulted in cell death, whereas Hsp90α knockout had little effect on cell survival and growth. Interestingly, the lack of Hsp90α selectively nullifies the tumour cells’ ability to invade *in vitro* and to form tumours in nude mice^6^. These findings suggest that Hsp90β is the long recognized intracellular ATPase-driven chaperone essential for life. In contrast, Hsp90α is dispensable for maintaining cell survival and homeostasis and its actual functions remained to be re-explored.

Only within the last decade have scientists uncovered previously unexpected cell surface-bound and secreted form of Hsp90α - collectively called extracellular Hsp90α (eHsp90α)^7^. Normal cells secrete Hsp90α under extracellular environmental stress, whereas many tumour cells, driven by activated internal oncogenes, constitutively secrete Hsp90α regardless the presence or absence of extracellular cues^8–14^. Hsp90α does not have the signal peptide for using the classical ER/Golgi protein trafficking pathway for secretion. Instead, proteomic and electron microscopic analyses first detected eHsp90α in cell-secreted exosomes, the smallest extracellular vesicles measuring between 30 and 150 nm in diameter^15–17^. Both *in vitro* and *in vivo* studies showed that eHsp90α has three cellular functions during wound healing and tumour progression: (i) anti-inflammation^18^, (ii) pro-survival by preventing cells from hypoxia-induced apoptosis^19^ and (iii) promoting cell migration^10,20^. To carry out these functions, eHsp90α acts as a *bona fide* extracellular stimulus that utilizes the following trans-membrane signalling pathway: binding to sub-domain II of low-density lipoprotein receptor-related protein-1 (LRP-1), transmitting the signal via the cytoplasmic NPVY motif of LRP-1, leading to activation of the Akt kinases^18,21^. Several monoclonal or recombinant antibodies against eHsp90α, 4C5^22^, scFv^10^ and 1G6-D7^6^ block secreted Hsp90α-mediated tumour cell invasion and metastasis in mice. Recent recent clinical studies reported dramatic elevations of the plasma Hsp90α protein levels in circulation in patients with breast, liver, lung, colorectal, and malignant melanoma cancers. Moreover, the variable plasma levels of Hsp90α closely correlate with the pathological stages of the cancers in these patients, making the plasma Hsp90α a new cancer diagnostic and therapeutic target^23–28^.

Secreted lipid-rich membrane vesicles, collectively called extracellular vesicles (EVs), have recently garnered a great deal of attention^29–32^. Based on differences in cargo composition, size, biogenesis and mechanisms of release, EVs are divided into three groups, apoptotic bodies, microparticles and exosomes. Of the three EV types, exosomes are the smallest in size and are believed to be formed as intraluminal vesicles inside early endosome-originated multivesicular bodies (MVB). The current model is that MVBs fuse with cell plasma membrane to release exosomes into the extracellular environment. Genomic and proteomic analyses revealed the profiles of exosome-associated molecules including genomic DNA, tRNA, mRNA, microRNA and cytoplasmic proteins from their host cells. It is believed that cells release exosomes for cell-to-cell communication under a wide variety of physiological and pathological conditions during development, host immune responses and tissue repair. Not surprisingly, this new and seemingly more efficient signalling mechanism between different types of cells has been taken advantage of by tumour cells during invasion and metastasis^33,34^.

To explore the therapeutic potential of eHsp90α in cancer, several questions remain to be answered. Is the loss of tumorigenicity in Hsp90α-knockout tumour cells due to blockade of the exosome secretion or loss of the extracellular functions of eHsp90α or both? Is eHsp90α inside the exosome lumen or on the external surface of exosomes, when it executes its functions? In the current study, we provide answers to these questions.

## Results

### The majority of eHsp90α protein is associated with tumour cell-secreted exosomes

Many tumour cells, including breast, colon, bladder, prostate, skin, liver and bone, constitutively secrete Hsp90α to gain motility and invasiveness *in vitro* and to support tumour formation and metastasis in mice^8,12,13^. We selected a tumour cell model to investigate the previously raised questions, based on the fact that the best-characterized oncogene that triggers Hsp90α secretion is hypoxia-inducible factor-1 alpha (HIF-1α), which is overexpressed in more than 50% of all invasive tumours in humans^35^. The human breast cancer cell line, MDA-MB-231, overexpresses HIF-1α and constitutively secretes Hsp90α^2,6,36^. In addition, we chose primary human dermal fibroblasts as the stromal cell model in this study.

**Figure 1:**
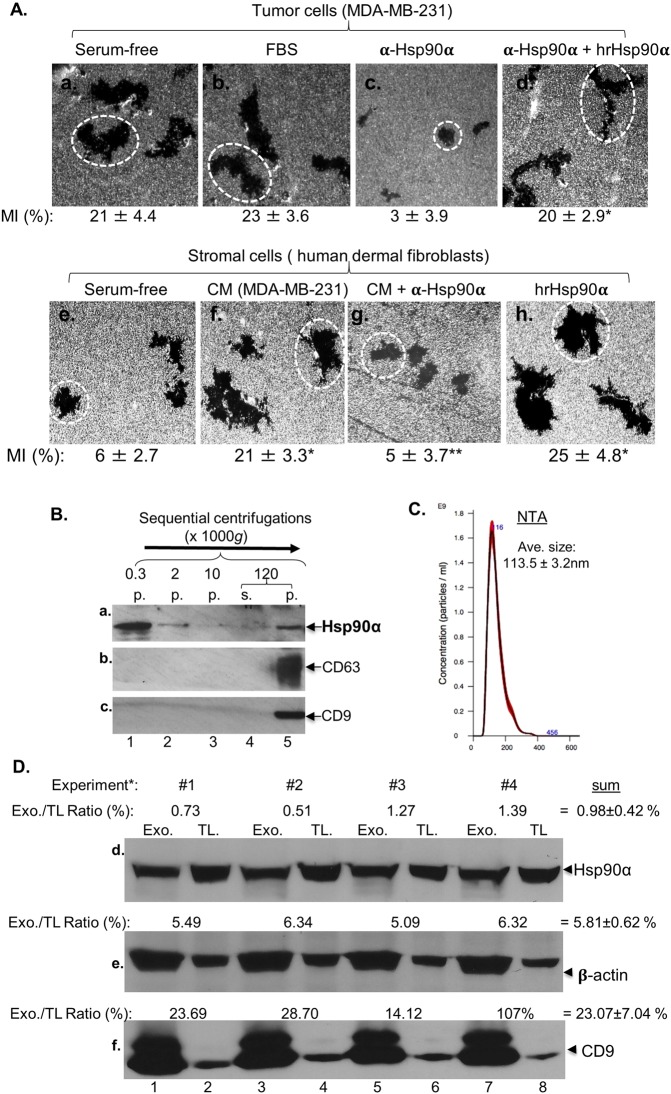
Tumour cells use exosome-associated eHsp90α to communicate with stromal cells. (**A**) Motility of parental MDA-MB-231 cells were analysed by colloidal gold migration assay and computer-assisted quantitation of 15 randomly selected images per condition as Migration Index (MI, %) (see details in Methods) under indicated conditions: 10% FBS, anti-Hsp90 antibody (30 μg/ml), hrHsp90α (10 μg/ml). Serum-free conditioned media (CM, 24 hours) of MDA-MB-231 cells were added to serum-starved human dermal fibroblasts with or without anti-Hsp90 antibody or hrHsp90α for similar migration assays. Representative cell migration images are shown. The experiment was repeated two more times. *Significant enhancement (*p* ≤ 0.05) against serum-free condition or antibody inhibition. **Significant inhibition by anti-Hsp90 antibody (*p* ≤ 0.05) against serum-free medium or CM treatment. (**B**) The tumour cell CM were subjected to four sequential centrifugations to collect pellet fractions (p) and the final supernatant fraction for immunoblotting analyses with indicated antibodies. (**C**) The 120,000 g pellet was resuspended in PBS and analysed by NTA. (**D**) The relative amounts of the three proteins in total lysate (TL) versus exosomes (Exo.). *TL sample loading: 2.3–1.5%, Exo. Sample loading: 100%. Intensity of bands from four independent experiments was quantitated by Image J software and the ratios calculated according to the percentage of sample loadings (see Methods).

To measure the constitutive motility of the tumour cells, we utilized the computer-assisted colloidal gold migration technique, which measures migration of individual cells and provides accurate quantitation of cell motility via the so-called “Migration Index” (MI, %) (Methods)^20,37,38^. As shown Fig. 1A, we found that MDA-MB-231 cells already showed a high basal motility under serum-free conditions (following 24-hour serum starvation) and little further enhancement in response to serum (panel b vs. panel a). This constitutive high motility was largely due to autocrine secretion of Hsp90α by the cells, since the addition of neutralizing anti-Hsp90α antibody, 1G6-D7, to the assay prevented the tumour cell motility (panel c vs. panel a). As expected, the antibody inhibition could be overcome by addition of excess^3x^ human recombinant (hr) Hsp90α protein (panel d). Computer-assisted quantitation of cell migration, i.e. MI (%), is shown below the representative microscopic images. The dotted circles represent the average size of the migration tracks under a given experimental condition.

To prove that tumour cells are secreting pro-motility factor, we collected conditioned medium (CM) of serum-starved tumour cells, concentrated^10x^ and added to migration assays with primary human dermal fibroblasts. We observed dramatically CM-stimulated cell migration (panel f vs. panel e), which could be inhibited the addition of mAb 1GD7 (panel g). The addition of hrHsp90α alone showed similar effect to CM on cell migration (panel h) Quantitative migration data as MI (%) are shown underneath each of the representative images.

We next examined whether eHsp90α is present in the CM and whether eHsp90α exists as a free molecule or associates with secreted EVs. We collected and subjected the CM of serum-starved MDA-MB-231 cells to the standardized protocol of low-(300 *g*), medium-(2,000 *g*), high-(10,000 *g*) and ultra-(120,000 *g*) speed centrifugations to obtain the individual pellet fractions of i) floating/dead cells, ii) cell debris, iii) large vesicles and iv) exosomes, as well as the final leftover supernatant (containing all soluble molecules) (Methods). As shown in Fig. 1B, Hsp90α was detected in the floating/dead cell pellet (lane 1) and, in a much smaller quantity, in the cell debris pellet (lane 2), but undetectable in the large vesicle pellet (lane 3) and the supernatant (lane 4). Instead, a significant amount of Hsp90α was enriched in the 120,000 g pellet fraction (panel a, lane 5) along with two well-characterized exosome markers, CD63 and CD9 (panels b and c, lanes 5), which were not detected in any of the lower centrifugation pellets (panels b and c, lanes 1 to 4). Furthermore, as shown in Fig. 1C, nanoparticle tracking analysis (NTA), which measures the size and the amount of EVs with a diameter range of 10–1000 nanometres (nm) in liquid suspension, of the 120,000 *g* pellet confirmed that the majority of the EVs was in the size range of exosomes (50–150 nm), with an average diameter of 113.5 ± 3.2 nm. These findings indicate that eHsp90α is not secreted as a “free” protein, but rather as a protein physically associated with the tumour cell-secreted exosomes. Finally, under quantitative comparisons of the exosome-associated versus the total cellular Hsp90α protein from four independent experiments (Methods), as shown in Fig. 1D, the exosome-associated Hsp90α accounts for approximately 1% of the total cellular Hsp90α, together with β-actin (~6%) and CD9 (~23%).

### Role of Hsp90α in regulation of exosome secretion and profiles of protein markers

**Figure 2:**
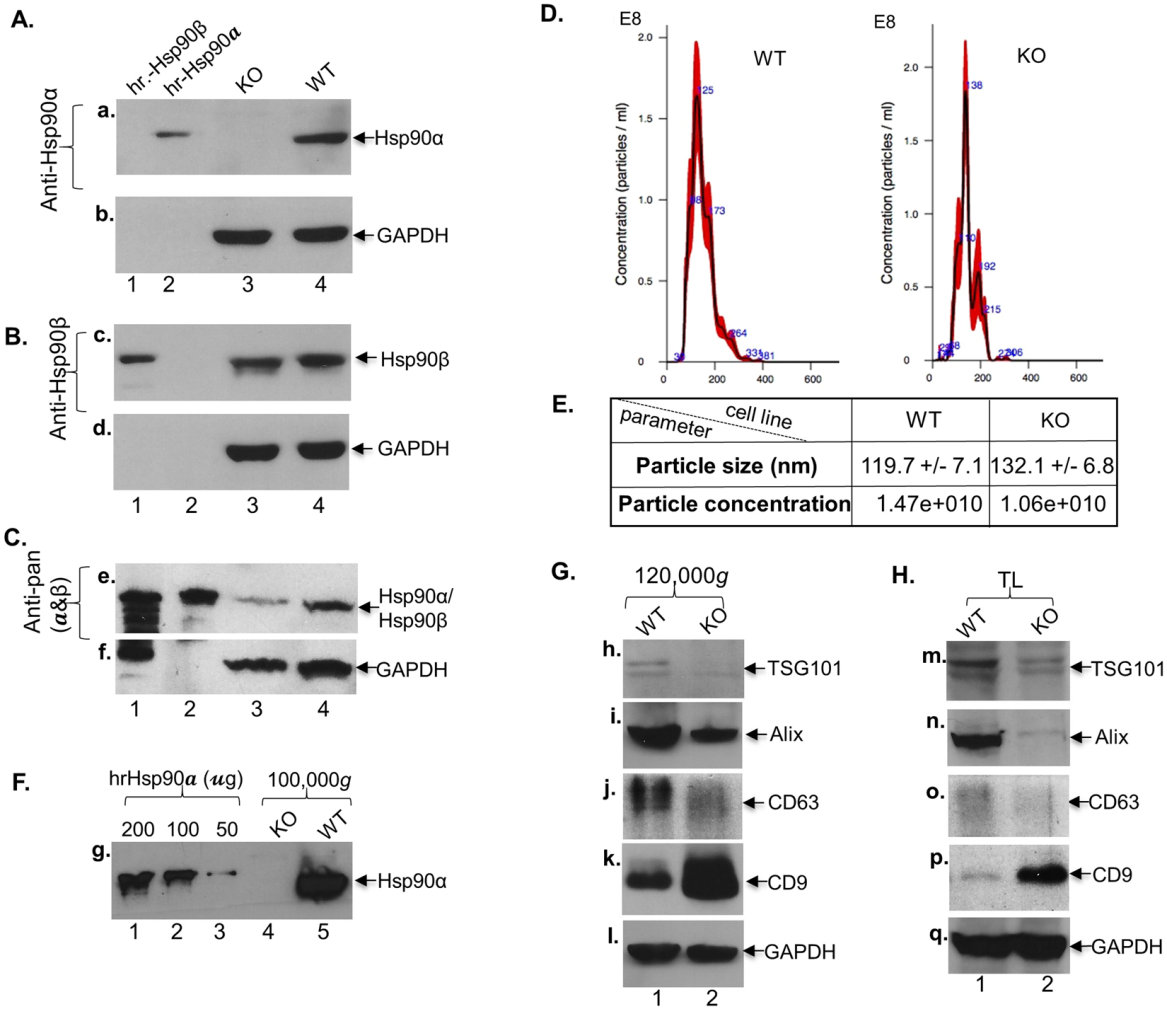
Effect of Hsp90α-knockout on exosome secretion and maker profiles. (**A**) CRISPR-cas9 knockout of Hsp90α in MDA-MB-231 cells, specifically the Hsp90α-KO clone #2 analysed here. Anti-Hsp90α antibody blot (panel a), with human recombinant (hr) Hsp90β (lane 1) and Hsp90α (lane 2) proteins as controls for antibody specificity. (**B**) Anti-Hsp90β antibody blot (panel c). (**C**) Anti-pan-Hsp90 antibody blot (panel e). (**D**) NTA analysis of the pellet fractions of 120,000 g ultracentrifugation of serum-free conditioned media from WT and Hsp90α-KO cells. (**E**) Table of quantitation of the NTA data, in which neither difference was statistically significant (p < 0.01) between WT and Hsp90α-KO cells. (**F**) Anti-Hsp90α antibody immunoblotting exosomes from WT and Hsp90α-KO cells (lane 4 vs. lane 5). (**G**) Immunoblotting of replicate exosome samples with the indicated antibodies. (**H**) Immunoblotting of the total cell lysates with the same set of antibodies. Each experiment was repeated for at least three times and similar results obtained.

The Hsp90 family proteins are best known as chaperones for intracellular signalling molecules^1,39^. A recent study reported that the single Hsp90 in *Drosophila* regulates membrane deformation and exosome release^40^. We studied whether Hsp90α is a regulator of exosome secretion or just a cargo molecule in secreted exosomes. To provide a definitive answer to these questions, we used CRISPR-cas9 technology to obtain Hsp90α-knockout (KO) MDA-MB-231 cells^6^. As shown in Fig. 2A, an anti-Hsp90α-specific antibody blot shows complete absence of Hsp90α in the Hsp90α-KO cells (panel a, lane 3) in comparison to the wild type (WT) cells (lane 4). The antibody only recognized the control hrHsp90α (lane 2), but not hrHsp90β (lane 1), protein. As shown in Fig. 2B, anti-Hsp90β antibody blot of a duplicate membrane shows equal amount of Hsp90β in both Hsp90α-KO and WT cells (panel c, lanes 3 and 4). Similarly, the anti-Hsp90β antibody recognized the control hrHsp90β (lane 1), but not Hsp90α (lane 2), protein. In contrast, as shown in Fig. 2C, anti-pan-Hsp90 antibody blot, recognized both hrHsp90α and hrHsp90β (panel e, lanes 1 and 2), showed a 50% reduction in the total amount of cellular Hsp90 proteins (panel e, lane 3 vs lane 4) (see additional evidence in Supplemental Information). We have previously shown that Hsp90β knockout led to few cell survival^6^.

Previous studies reported mixed and sometimes conflicting results on whether exosome markers, such as CD63, TSG101, STAM1 and Hsp90, play a role in exosome biogenesis and secretion^31,32,41,42^. When isolated exosomes from either WT or Hsp90α-KO cells were subjected to NTA analysis, we were surprised to find that Hsp90α knockout had little effect on either the distribution or the amounts of exosomes secreted by the cells. As shown in Fig. 2D, the size distribution (x-axis) and the concentrations (y-axis) of the exosomes collected from Hsp90α-KO and WT cells were comparable (actual numeric values are shown in Fig. 2E). When we subjected the exosome fractions from an equal number of WT and Hsp90α-KO cells to Western blot analysis, as shown in Fig. 2F, eHsp90α was completely absent from exosomes isolated from the Hsp90α-KO cells (lane 4), unlike exosomes isolated from the WT cells (lane 5). hrHsp90α protein was included as the positive control (lanes 1–3). However, as shown in Fig. 2G, Hsp90α KO showed differential effects on the levels of other exosome markers: a significant decrease in three markers, TSG101 (panel h), Alix (panel i) and CD63 (panel j), a dramatic increase in CD9 (panel k) and little change in GAPDH (panel l). We also detected corresponding changes in the cellular levels of these markers (panels m to q). Therefore, while Hsp90α differentially regulates cellular levels of exosome markers, neither Hsp90α KO nor changes in the levels of these exosome markers affected the total amount of exosome secretion.

### eHsp90α-free exosomes lost a key communication signal between tumour and stromal cells

**Figure 3:**
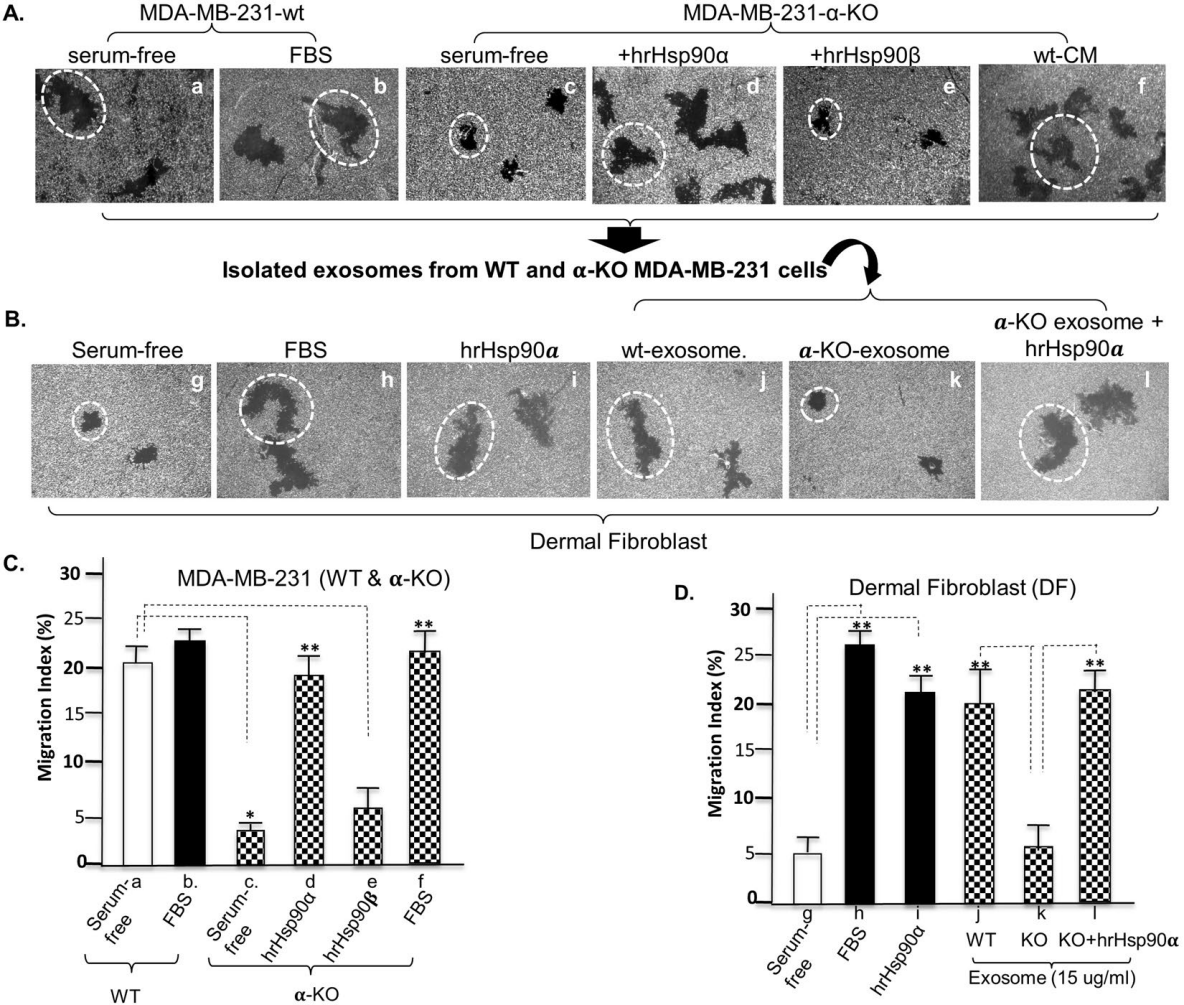
Hsp90α-knockout exosomes fail in tumour-to-stromal cell communication. (**A**) WT and Hsp90α-KO cells were subjected to the colloidal gold motility assay with indicated treatments, and representative cell migration images are shown. (**B**) The 120,000 *g* pellet (exosome) fractions were isolated from serum-free CM of WT and Hsp90α-KO cells and tested for their ability to stimulate human dermal fibroblast cell migration with or without exogenous Supplementation with hrHsp90α. (**C**,**D**) The above cell migration data were quantitated by a as Migration Index (MI, %) (Methods). This experiment was repeated more than four times. *Significant inhibition by Hsp90α knockout (*p* ≤ 0.05) against the WT cell under serum-free medium. **Significant enhancement (*p* ≤ 0.05) by exogenously added exosomes, hrHsp90α protein, or FBS against serum-free condition.

We next tested if eHsp90α is essential for both the constitutive motility of tumour cells themselves and the ability of the tumour cells to communicate, via secreted exosomes, with stromal cells. As expected, as shown in Fig. 3A, the Hsp90α-KO tumour cells lost the constitutive motility under serum-free conditions (panel c vs. panel a). This defect could be fully rescued by the addition of hrHsp90α (panel d vs. panel c), but not hrHsp90β (panel e), protein to the assay. We then tested the exosomes isolated from the WT or the Hsp90α-KO tumour cells for stimulation of migration of normal stromal cells. As shown in Fig. 3B, under serum-free conditions, human dermal fibroblasts showed a basal level of motility (panel g). As expected, serum or hrHsp90α stimulation dramatically enhanced stromal cell migration (panels h and i). Exosomes isolated from WT MDA-MB-231 cells strongly stimulated the stromal cell migration (panel j), but not from the Hsp90α-KO cells (panel k). However, Supplementation of hrHsp90α protein to exosomes of Hsp90α-KO cells fully restored the effect on promoting the stroma cell pro-motility (panel l). Computer-assisted quantitation of cell migration as MI (%) for both experiments, as shown in Fig. 3C,D, indicated that eHsp90α is responsible for dissemination of the migratory signal from tumour cells to stromal cells.

### Tumour-secreted eHsp90α is on the external surface of exosomes

**Figure 4:**
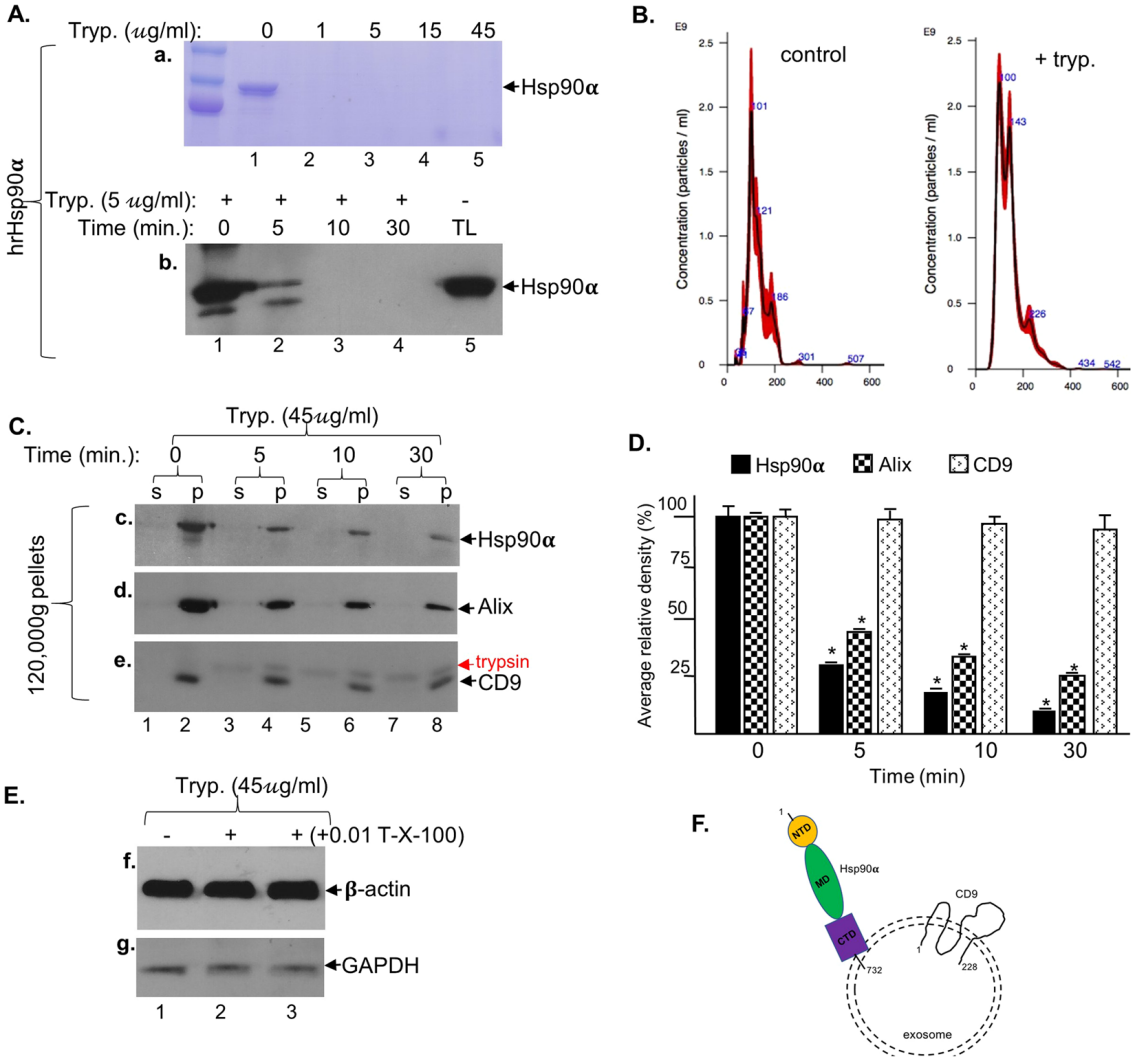
Exosome-associated Hsp90α sensitive to trypsin digestion. (**A**) Dose- and time- dependent digestion of hrHsp90 protein (2 μg) by TPCK-treated trypsin. (**B**) Exosomes after digestion (45 μg/ml trypsin, 30 min at 37 °C), were re-centrifuged at 120,000 g to re-collect exosomes and analysed by NTA. (**C**) Time course of trypsin digestion of isolated exosomes, followed by re-collecting the 120,000 *g* pellets and immunoblotting analyses with indicated antibodies. (**D**) Intensities of the bands quantitated by Image J. This experiment has been repeated three times. *Significant reduction in reference to no trypsin control, *p* < 0.05. (**E**) Trypsin failed to affect exosome-associated β-actin or GAPDH even in the presence of Triton-X-100. (**F**) A hypothetical presentation of surface-bound Hsp90α and CD9 to explain the differential sensitivities of Hsp90α and CD9.

**Figure 5:**
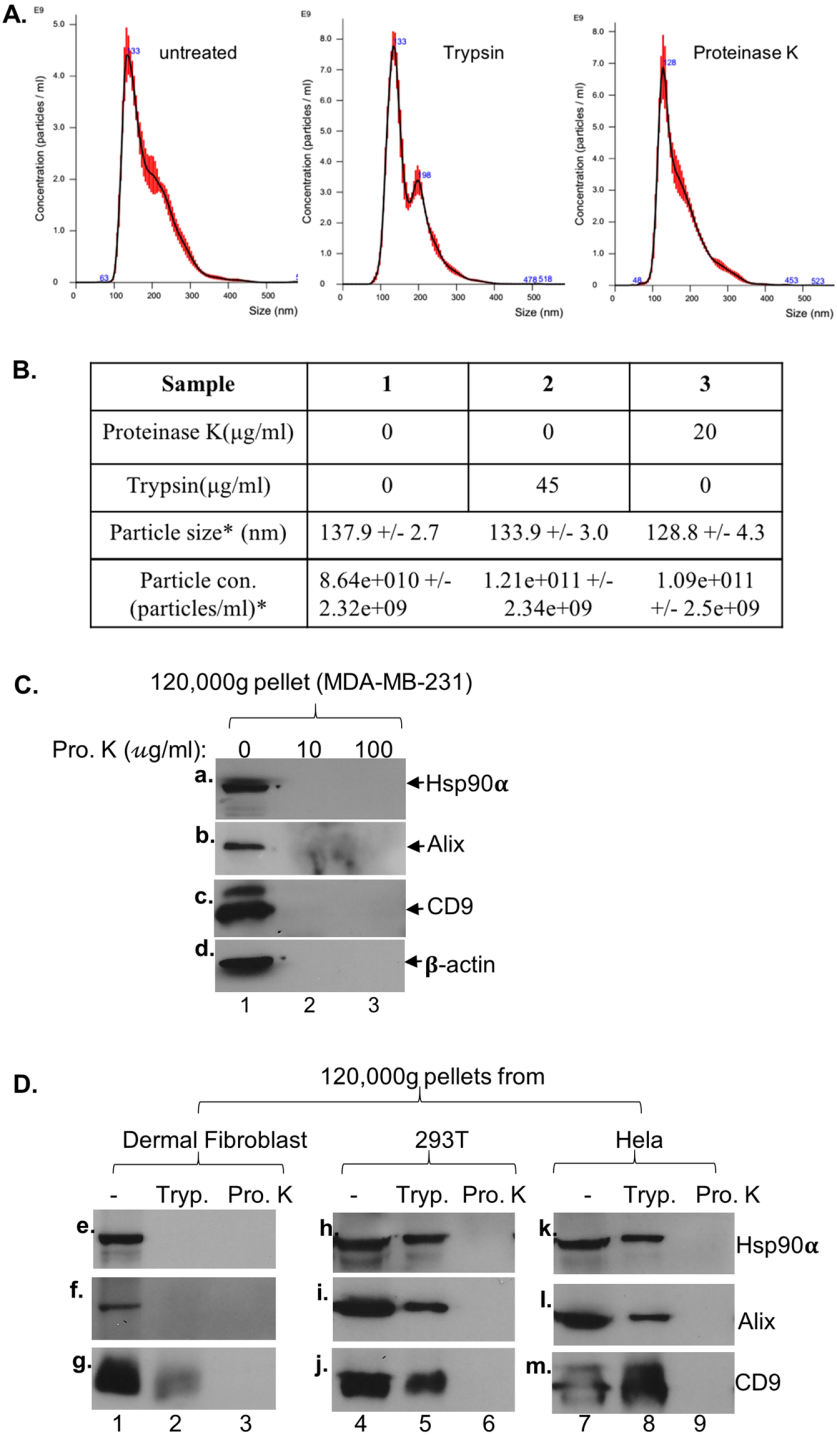
Comparisons between trypsin and proteinase K Hsp90α on exosomes from various normal and cancer cell lines. (**A**) Our conditions of neither trypsin nor Proteinase K digestion affect the exosome integrity, as shown by NTA. (**B**) Quantitation of the NTA data, which does not show statistically significant difference in distribution and relative amounts between untreated and treated exosomes, *p* < 0.01. (**C**) Tumour cells exosome subjected to Proteinase K digestion (20 μg/ml, 60 min, 37 °C), followed by immunoblotting analyses. (**D**) Exosomes were isolated from additional three human cell lines, dermal fibroblasts, 293 T and Hela, and subjected to trypsin (45 μ mg/ml, 30 min, 37 °C) or Proteinase K (20 μg/ml, 60 min, 37 °C) digestion and subjected to immunoblotting analyses with indicated antibodies. These experiments were repeated multiples times with similar results.

Purified hrHsp90α protein in the absence of any carriers promotes cell migration by binding, activating and signalling through the LRP-1 cell surface receptor on both normal and tumour cells^6,20,37^. Since tumour cell CM promotes cell migration via exosome-associated eHsp90α, we predicted that exosomes must carry Hsp90α on their external surface to execute this function. To investigate this hypothesis, we carried out protease analyses, first with trypsin. As shown in Fig. 4A, two micrograms of hrHsp90α protein were completely digested even by the lowest concentration of trypsin (1μg/ml) (panels a) (Note: Commercial trypsin solutions for cell culture contain at least 0.05% or 500μg/ml of trypsin). The digestion completed within 10 minutes (panel b). Total cell lysates (TL) were included as the control. Following digestion, duplicate reaction samples were subjected to 120,000 g ultracentrifugation and the pellets were re-suspended for NTA analysis. As shown in Fig. 4B, even after the strongest experimental conditions (45 μg/ml of trypsin for 30 min at 37 °C in PBS buffer), the amount and overall distribution of the exosomes remained unaffected. Therefore, we used this condition for isolated exosomes. Exosomes isolated from the tumour cells were subjected to a time course of trypsin digestion. The reactions were stopped and re-subjected to 120,000 g ultracentrifugation. Both pellet and supernatant fractions were collected, equalized to the same volume (by concentrating the supernatant) and subjected to Western immunoblotting analysis. As shown in Fig. 4C, a time-dependent decrease in exosome-associated eHsp90α (panel c) and Alix (panel d) was observed. Surprisingly, the levels of the well-known exosome surface-bound tetraspanin, CD9, remained almost unchanged (panel e). As expected, none of the exosome markers were detected from the supernatants of the reactions, confirming integrity of the exosomes. Quantitation of the protein bands by Image J analysis is shown in Fig. 4D, which showed ~80%, ~70% and less than 10% reduction of Hsp90α, Alix CD9, respectively. These findings are consistent with a previous report by McCready *et al*. that exosome-associated Hsp90alpha is easily accessible to proteases^12^. Under the similar conditions, however, trypsin was unable to digest exosome-associated β-actin (Fig. 4E, panel f, lane 2) or GAPDH (panel g, lane), even in the presence of Triton-X-100 (lanes 3). Our interpretation of the trypsin digestion results is schematically shown in Fig. 4F, in which the majority of the eHsp90α polypeptide is exposed to the extracellular environment and has more sites for trypsin digestion. Whereas only approximately 40% of the CD9’s 228-amino acid peptide is facing outside. Therefore, we replaced trypsin with Proteinase K, a broad-spectrum serine protease that cleaves peptides after hydrophobic amino acids. Figure 5A demonstrates that Proteinase K digestion (panel c), like trypsin (panel b), did not affect the distribution and the amounts of exosomes (panel a), as quantitated in Fig. 5B. However, as shown Fig. 5C, in contrast to trypsin treatment Proteinase K was able to achieve a complete removal of not only Hsp90α (panel a), Alix (panel b), and CD9 (panel c), but also β-actin (panel d). The cause for this rather surprising observation will be discussed.

These findings are not cell-type specific. Exosomes were isolated from additional three types of cells of human origin, dermal fibroblasts, 293 T and Hela cells and subjected to both trypsin and Proteinase K digestion. As shown in Fig. 5D, in dermal fibroblasts, all exosome-associated markers were equally sensitive to both trypsin and proteinase K (panels e to g). In contrast, the same exosome protein markers from 293 T and Hela cells were significantly less sensitive to trypsin digestion under the same conditions (panels h-j, lanes 5 and panel k-m, lanes 8). These surprising results suggest that the same protein markers may locate differently in exosomes from different cell types. Again, however, proteinase K treatment eliminated all four markers from the exosomes of the three cell types (lanes 3, 6 and 9). Taken together, these findings challenge the current model of exosome biogenesis and secretion^16,17,29–32^, according to which cytosolic proteins, such as Hsp90α and Alix, cannot not appear on the external membrane of ILVs or exosomes. Even more surprisingly, we found that exosome-associated β-actin was removed by Proteinase K. Likewise, seven previous publications all showed different results on a dozen of exosome markers after Proteinase K treatment under almost identical conditions. The reason for such a huge discrepancy from different laboratories remains unknown.

### Monoclonal antibody, 1G6-D7, against eHsp90α blocks exosome-mediated communication from tumour cells to stromal cells

**Figure 6:**
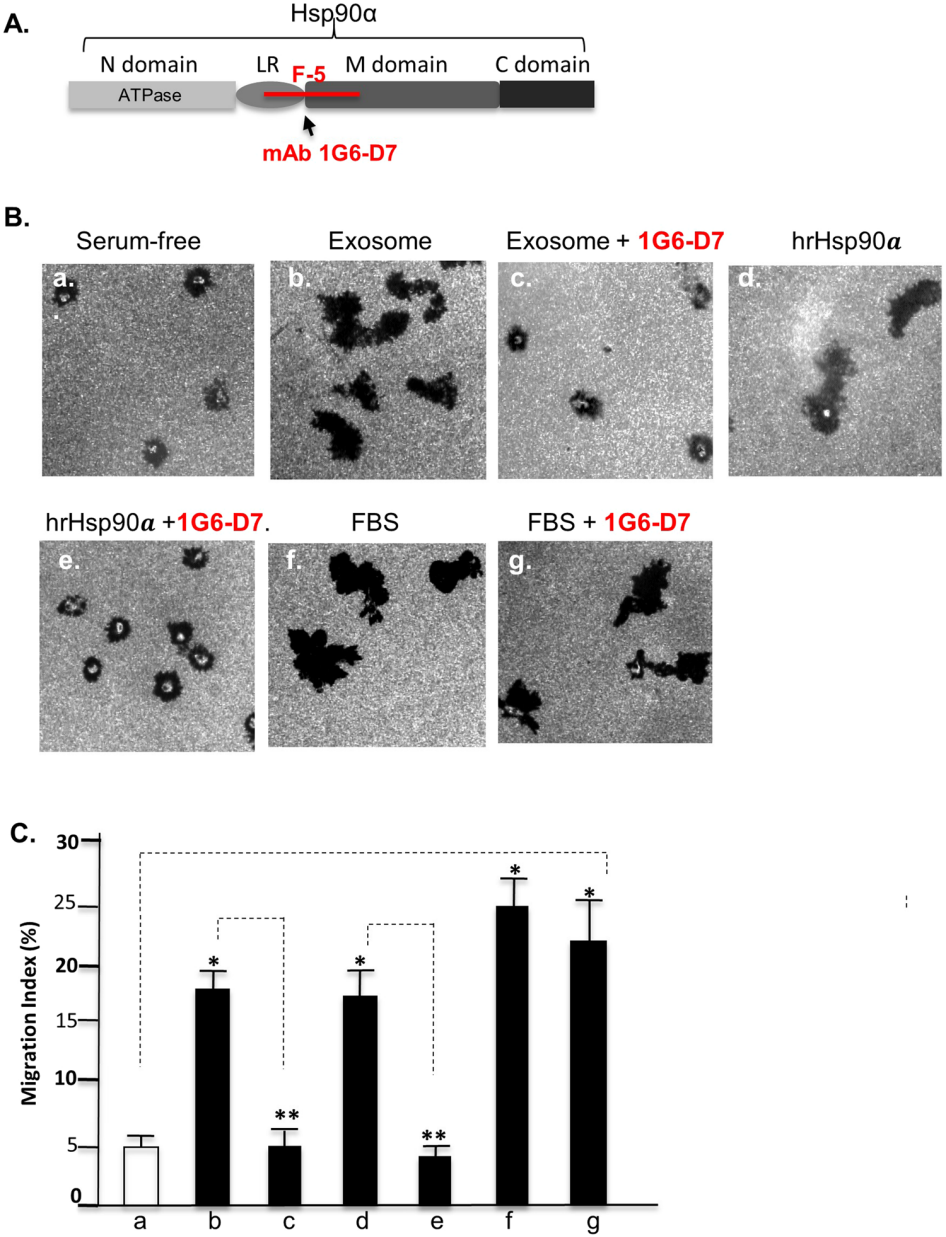
mAb 1G6-D7 blocks tumour cell-secreted exosomes to communicate with stromal cells. (**A**) Schematic representation of the recognition site by mAb 1G6-D7 within the F-5 region of human Hsp90α^6^. (**B**) mAb 1G6-D7 blocks the promotion of human keratinocyte migration by tumour-secreted exosomes. In this experiment, 5 μg/ml exosomes, 15 μg/ml mAb 1G6-D7, 10 μg/ml hrHsp90α and 10% FBS were used. (**C**) Computer-assisted quantitation of the cell migration from B as Migration Index (%). *Significant enhancement (*p* ≤ 0.05) by exosomes, hrHsp90α, or FBS on cell migration against the serum-free condition. **Significant inhibition by 1G6-D7 (*p* ≤ 0.05) against exosome or hrHsp90α-stimulated cell migration.

If tumour-secreted exosomes carry eHsp90α on their external surface, we anticipated that an anti-eHsp90α neutralizing monoclonal antibody would block the communication from tumour-secreted exosomes to stromal cells, since antibodies do not penetrate the lipid membrane of exosomes. We tested the monoclonal, 1G6-D7, which was recently developed in our laboratory^6^. As schematically shown in Fig. 6A, 1G6-D7 targets the “active site”, so-called F-5 region, within eHsp90α. We purposely chose a second primary human cell type, keratinocytes, to prove our hypothesis. As shown in Fig. 6B, the tumour-secreted exosomes strongly stimulated human keratinocyte migration (panel b vs. panel a). However, the addition of 1G6-D7 to the assay completely nullified the pro-motility activity of the exosomes (panel c vs panel b). The effectiveness of 1G6-D7 was confirmed by its complete inhibition of hrHsp90α-induced cell migration (panel e vs. panel d). The specificity of 1G6-D7 was shown by its lack of inhibition of serum-induced cell migration (panel g vs. panel f). The migration data were subjected to computer-assisted quantitation and statistical analysis, as shown in Fig. 6C. This finding provides direct support that tumour-secreted Hsp90α is located on the exosome surface facing the extracellular environment.

## Discussion

**Figure 7:**
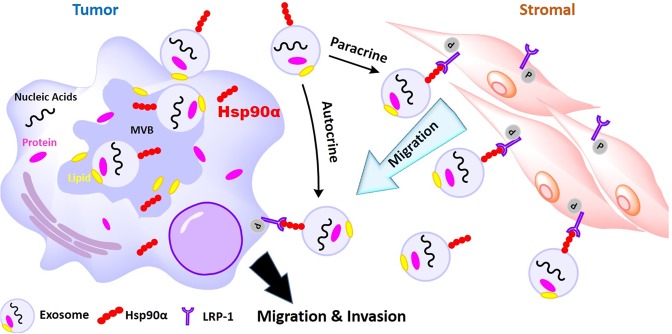
A working model for tumour-secreted and exosome-bound Hsp90α. Exosome biogenesis recruits cytosolic Hsp90α to external surface of exosomes, via a currently unknown mechanism, inside the cell. Upon secretion, the exosomes drive, via the surface-bound Hsp90α, both tumour and stromal cell migration via both autocrine and paracrine mechanisms. The “moved-in” stromal cells aid better survival and invasion of the tumour cells. This intercellular communication is thought to play a role in tumour cell invasion and metastasis. Therefore, eHsp90α on external surface of tumour-secreted exosomes presents as an attractive target for anti-metastasis therapeutics.

During the past decade, studies of the Hsp90 family proteins, especially Hsp90α, have uncovered a new paradigm – the non-chaperone, ATPase-independent, extracellular role of Hsp90α - in wound healing and tumour progression. Unlike the essential need for Hsp90β in cell survival and animal development, Hsp90α shows a dispensable role as an intracellular chaperone for cell survival in culture and animal development. Instead, its main functions appear to be carried out by eHsp90α in response to in environmental stress^8,12,13^. Hsp90α cannot replace Hsp90β to fulfil the intracellular chaperoning duties. Likewise, Hsp90β has shown limited extracellular actions. Our laboratory has demonstrated that this difference is determined at least by two evolutionarily conserved amino acids, lysine-270 and lysine 277, in all Hsp90α sub-family members and glycine-262 and threonine-269, in all Hsp90β sub-family members. Swapping these two amino acids between Hsp90α and Hsp90β, as expected, nullifies the eHsp90α function and makes Hsp90β act like eHsp90α, respectively^6^. In this current study, we have advanced the mechanism of action by eHsp90α by demonstrating that the majority of eHsp90α is associated with secreted exosomes. While Hsp90α does not participate in regulation of the exosome secretion step, eHsp90α-free exosome lost its ability to conduct the important intercellular communication from tumour cells to stromal cells – promotion of cell motility. Most importantly, we provide evidence that the exosome-associated eHsp90α resides on the external surface of exosomes facing the extracellular microenvironment. Protease treatment and monoclonal antibody inhibition removes the physical presence in exosomes and nullifies the function of eHsp90α, respectively. These findings provide a long-sought-after explanation for how exosome-associated proteins execute their functions from extracellular microenvironment. A schematic representation of these findings is depicted in Fig. 7, in which tumour cells use secreted exosomes to attract stromal cells via eHsp90α on the exosome surface. This tumour-stromal cells interaction supports the tumour cell invasion and metastasis.

The current exosome biogenesis and secretion models do not explain how host cytoplasmic proteins could possibly locate on the external surface of secreted exosomes. First, regarding surface-bound and luminal protein makers in exosomes, studies from different laboratories showed dramatic variations even under seemingly identical experimental conditions^43–48^. Regarding what exosome protein markers are sensitive or resistant to Proteinase K digestion, for example, the reported results raised more questions than answers. Lee *et al*. reported that exosome-associated ICAM-1 is sensitive and β-actin is resistant to Proteinase K treatment^43^. Cvjetkovic and colleagues showed that most EV/exosome markers were resistant or partially resistant to Proteinase K, except GAPDH and STUB1^44^. Melo *et al*. reported that TSG101 and CD9 could be removed from exosomes by Proteinase K only in the presence of Triton-X-100^45^. Similarly, Banfer and colleagues showed that Alix and Galetin-3 could survive Proteinase K digestion in the absence, but not presence, of Triton-X-100^46^. Jeppesen *et al*. isolated exosomes and subjected it to fractionation of membrane and soluble fractions. They showed that CD63, Akix and CD9 were in the membrane, whereas T24, FL3 and SLT4 in the soluble, fraction^47^. Phoonsawat *et al*. showed that Proteinase K removes CD63 from exosomes even in the absence of Trito-X-100^48^. We showed that Proteinase K digestion under similar conditions did not affect the integrity of exosomes, but it was able to remove most of the exosome markers from exosomes. While the reason for this wide-range discrepancy is unclear, it suggests that we still do not have a full understanding about the nature of association between exosomes and their protein markers. According to current models, cytosolic proteins should reside in the luminal or inner aqueous membrane surface of ILV or exosomes, and they could not possibly face the external or extracellular surface^31,32^. A plausible explanation is that a pool of the cytoplasmic Hsp90α protein is first translocated to the outside surface of the plasma membrane prior to the inward budding and formation of early endosomes, so that they could appear at the external surface of secreted exosomes.

Our calculations showed that the amount of exosome-associated Hsp90α protein accounts for approximately 1% of the total cellular Hsp90α protein in MDA-MB-231 tumour cells. While it is hard to characterize the “1%” as high or low at its face value without a relevant context, a physiologically relevant question is whether the amount of exosome-associated Hsp90α could reach the lowest threshold functional concentration. Taking a 15-cm cell culture dish with a confluent cell monolayer as an example, the size of the cell pellet following centrifugation is ~0.2 ml^3^. That is to say that the ~0.2 ml^3^ cell body represents by and large the living space of these cells *in vivo*. Therefore, the actual “fluid” surrounding the cell population *in vivo* should even be smaller. Based on these calculations, the liquid environment of these cells should be hundreds of times less than the 20 ml of culture medium used to cover the cell monolayer in the 15-cm cell culture dish. If we concentrate the 20 ml of conditioned medium to the presumed volume of the liquid environment *in vivo* and take the lowest amount of hrHsp90α detection by the anti-Hsp90α antibody as 0.05–0.1μg by Western^19^, the amount of exosome-associated Hsp90α (in Fig. 1D) could reach the reported functional concentration of micrograms per ml for hrHsp90α protein^20^. Therefore, we suggest that the 1% exosome-associated Hsp90α protein is physiologically relevant.

Finally, findings of this study provide a potentially new anti-tumour therapeutic approach by inhibiting the exosome surface-bound Hsp90α. For instance, it is known that a major regulator of the exosome-mediated Hsp90α secretion is hypoxia-inducible factor-1alpha (HIF-1α), whose overexpression and activation have been reported in 50 to 100% of all invasive tumours in humans^34^. While directly targeting HIF-1α in tumours so far has been unsuccessful, inhibiting the HIF-1α-driven and tumour-secreted Hsp90α may represent an alternative approach. As mentioned previously, clinical studies showed that the increased plasma Hsp90α levels in patients with a wide variety of cancers closely correlate with the pathological stages of the cancers^23–28^. Therefore, injection of anti-Hsp90α antibodies to the circulation could potentially become a new treatment for a broad spectrum of human cancers.

## Materials and Methods

### Cell lines

Limited-passage stocks of the human triple negative breast cancer cell line, MDA-MB-231, were obtained from liquid nitrogen storage from Dr. Michael Press (University of Southern California, Los Angeles) and were cultured in DMEM medium with high glucose Supplemented with 10% FBS (Thermo Scientific, MA, USA). Primary human dermal fibroblast, Hela cells and 293 T cells were cultured in DMEM with high glucose and 10% FBS (Thermo Scientific, MA, USA). Primary human keratinocytes were cultured in EpiLife medium with added growth factor Supplements (Thermo Scientific, MA, USA). The third or fourth passages of the primary human cells were used for this study.

### Antibodies and reagents

Anti-CD63 (EXOAB CD63-A1), anti-CD9 (13403), and anti-CD81 (EXOAB CD81A-1) antibodies were purchased from System Biosciences (Mountain View, CA) and Cell Signalling Technology (Danvers, MA), respectively. Anti-TSG101(sc-7964) antibody was obtained from Santa Cruz Biotechnology (Santa Cruz, CA). Anti-Alix antibody was received as a gift from Dr. F. T. Liu (University of California at Davis). Mouse monoclonal antibodies against human Hsp90α (CA1023) and human Hsp90β (SMC107) were purchased from Calbiochem (Billerica, MA) and Stressmarq Biosciences (Victoria, BC, Canada), respectively. The mouse monoclonal antibody, 1G6-D7, against both Hsp90α and Hsp90β was established in our laboratory^6^. Anti-GAPDH (GTX28245) antibody was obtained from Genetex (Irvine, CA). TPCK-treated Trypsin was purchased Sigma (St Louis, MO). Proteinase K (AM2546) was purchased from Thermo Fisher Scientific (Waltham, MA).

### CRISPR/Cas9 knockout Hsp90α (Gene ID:3320) and human Hsp90β (Gene ID:3326) in MDA-MB-231 cells

Please see detailed procedures by Zou *et al*.^6^. In the current study, we verified and focused on clone #2 of the Hsp90α-knockout MDA-MB-231 cell line.

### Exosome isolation and analyses

Cells in approximately 80% confluency were washed in warm PBS and further incubated in pre-warmed serum-free DMEM for an additional 72 hours. Exosomes were isolated from conditioned medium (CM) of the cells by using the technique of sequential centrifugations^29^. Centrifugations at 300 g (10 min), 2,000 g (10 min), 10,000 g (30 min) at 4 °C eliminate dead cells, cellular debris and microparticles from CM, respectively. Following each centrifugation, the pellet was re-suspended in PBS and saved for later analysis, and the supernatant was subjected to ultracentrifugation. After the final ultracentrifugation in Beckman’s SW41Ti rotor at 120,000 *g* for 90 min at 4 °C, the supernatant was removed and saved. The pellet was re-suspended/washed in 10 ml of cold PBS and re-centrifuged at 120,000 × g for an additional 90 min to remove any non-specifically bound proteins. The PBS was removed and discarded. The pellet was finally re-suspended in 100 ~ 300 μl fresh PBS. The size distribution and concentration of the isolated exosomes were analysed using the NS300 nanoparticle tracking analysis (NTA) device (NanoSight, Malvern Instrument) equipped with a 532 nm green laser. The camera operates at 30 frames per second (fps), capturing a video file of the particles moving under Brownian motion. All settings of camera remained unchanged during measurements. The NTA 3.1 software tracks many particles individually and using the Stokes-Einstein equation calculates their hydrodynamic diameters.

### Protease digestions

Approximately 5 μg of isolated exosomes from CM of various cells were re-suspended in final volume of 0.5 ml of PBS with indicated concentrations of trypsin or proteinase K for 30 or 60 min, respectively, at 37 °C with moderate agitation (Thermomixer 5436, Eppendorf). Recombinant proteins were used as controls as indicated. At end of the reactions, exosome samples were diluted to 10 ml with PBS and subjected to 120,000 *g* centrifugation. The pellets were collected and analysed by immunoblotting analyses with indicated antibodies.

### Western immunoblotting analysis and quantitation

Presence of exosome markers, including CD63, CD9, Alix, TFG101 and Hsp90a, were verified using Western immunoblotting analyses. Cellular lysates and exosomal proteins (~5 μg) were separated on SDS-PAGE and transferred onto PVDF membranes. The procedure of Ponceau Red staining was used for confirming protein transfer. The primary antibodies against the following proteins were used for the analysis: Hsp90α (1:1000), CD9 (1:1000), CD63 (1:20), TSG101 (1:20), Alix (1:5000), and GAPDH (1:1000). Secondary anti-rabbit IgG (1:10,000) and anti-mouse IgG (1:10,000) were used as instructed. The band intensity in Western blotting was quantified using Image J software (National Institutes of Health) via the following procedure: digital images of radiograph films were opened and converted to grayscale. Using a rectangular selection tool, a rectangle was drawn which contained all Western blot protein bands incubated with the same primary antibody from the same experiment. Next, “Plot lanes” was selected from the “Analyze” menu to create a profile plot of all the bands. Lines were then drawn between the peaks that represented darker bands. All measurements were recorded for the highlighted peaks. The peak of the control sample was selected as the standard and then the relative density of the peaks of the other bands was calculated in reference to the control. Statistical significance was determined using a two-tailed Student’s t-test and one-way ANOVA. Statistical significance was accepted when p < 0.05.

### Estimation of the percentage of exosome-associated Hsp90α, in reference to CD9 and β-actin

In order to clearly detect and estimate the amount of exosome-associated proteins by using SDS-PAGE and Western immunoblot, one would need the total amount of secreted exosomes from at least several millions of cells (e.g. a confluent 15-cm cell culture dish). Meanwhile, it is not possible to load the entire total lysates of the cells next to the exosome sample on the gel. Therefore, our approach, as shown in Fig. 1D, was to load a smaller and known percentage of the totally lysates together with the 100% of the isolated exosomes, so that the Western blotting bands of the total lysates will not be overexposed and quantifiable by imaging. Through a series of pre-runs, we chose the range of 1.5–2.3% of the total lysates, depending upon each of the four independent experiments. The numbers of the band intensity from total lysates by Image J were then converted (increased) to their presumed 100% scales. The final percentage for given protein in exosomes was calculated as: band intensity (from exosome)/corrected band intensity (total lysate) x 100% from means of multiple independent experiments.

### Computer-assisted colloidal gold migration assay and data quantitation

The colloidal gold migration assay was initially described by Albrecht-Buehler^37^ and modified by our laboratory using computer-assisted analysis^36^. Briefly, glass coverslips (35 mm in diameter) were pre-coated with 1% freshly prepared bovine serum albumin (BSA) in phosphate-buffered saline (PBS), dried by air, and placed into 12-well tissue culture plates with one coverslip per well. Colloidal gold chloride solution (colloidal gold chloride suspension (6.85 mg/ml H_2_O:30% Na_2_CO_3_:H_2_O, 0.9 ml:3 ml:5.5 ml) was heated in an 50-ml Erlenmeyer flask with constant swirling until boiling and then removed from the heat source. An equal volume of freshly prepared 0.1% formaldehyde solution was slowly added to the gold salt mixture with swirling. The mixture (when turning to purple-brown) was immediately plated at 1 ml/well into the 12-well plates with coverslips and left undisturbed for 2 h to let the colloidal gold particles settle on the BSA-coated coverslips. The supernatant was removed and the wells gently rinsed once with 1 ml of Hanks’ balanced salt solution (HBSS) without Ca^2+^. 1 ml of HBSS containing 5 mM Ca^2+^ and 20 μg/ml of native rat tail type I collagen was added and then incubated at 37 °C for 2 h. Unattached collagen molecules were removed and wells were rinsed once with HBSS without Ca^2+^. Uncoated areas of the colloidal gold, if any, were blocked by incubating with 0.1% BSA.

Serum-starved (0.2% FBS for 24 hours) cells were trypsinized, suspended, and counted. Three thousand cells/well were plated and allowed to migrate for 16 h. Cell migration was stopped by addition of 0.1% formaldehyde in PBS for 10 min. Individual cell migration was visualized under a dark field microscope that is linked to a computer via a real-time charge-coupled device camera (KP-MIU; Hitachi Denshi, Woodberry, NY). Fifteen of two to three single cell track-containing fields under each condition were randomly selected, photographed and analysed by the computer using NIH Image 1.6 software, which gives rise to the migration index (MI). The MI represents the percentage (%) of the field area consumed by cell tracks produced by migrating cells over the total field area under the microscope x 100. Finally, it is noticed that different cell types show variable basal levels of motility. Moreover, the quantitative data of the colloidal gold migration assay may vary from experiment to experiment, depending upon 1) BSA coating quality, 2) plated colloidal gold density, and 3) collagen coating and cell culture conditions. Therefore, the “relative” migration indices under different conditions are only valid within a same experiment, but the actual numbers may not be comparable cross independent experiments.

### Statistical analysis

All numerical results are reported as mean and standard deviation (s.d.). The band intensity in Western blotting was quantified with Image J software (National Institutes of Health). Statistical significance was determined using a two-tailed Student’s t-test and one-way ANOVA. Statistical significance was accepted when *p* < 0.05. The methodology to determine whether the differences in MIs between experimental sets of migrating cells are significant has been published by us^19^. Final presentation as mean ± s.d. was based on at least three independent and corroborating experiments. Confirmation of a difference in migration as statistically significant requires rejection of the null hypothesis of no difference between mean migration indices obtained from replicate sets. A *p* value equal or less than 0.05 was considered statistically significant.

## Supplementary information

raw data set
