# Supplementary material for: Tumour-Secreted Hsp90α on External Surface of Exosomes Mediates Tumour - Stromal Cell Communication via Autocrine and Paracrine Mechanisms
Source: Sci Rep. 2019 Oct 22;9:15108. doi: 10.1038/s41598-019-51704-w (PMC6805946; doi:10.1038/s41598-019-51704-w)

Tumour-Secreted Hsp90α on External Surface of Exosomes Mediates Tumour - Stromal Cell Communication via Autocrine and Paracrine Mechanisms

Xin Tang [1,3], Cheng Chang [1,3], Jiacong Guo [1], Vadim Lincoln [1], Chengyu Liang [2], Mei Chen [1], David T. Woodley[1], and Wei Li [1,4]

[1]Department of Dermatology and [2]Departments of Molecular Microbiology & Immunology, the Norris Comprehensive Cancer Centre, University of Southern California Keck Medical Centre, Los Angeles, CA 90033, USA

Original films for Figure 1

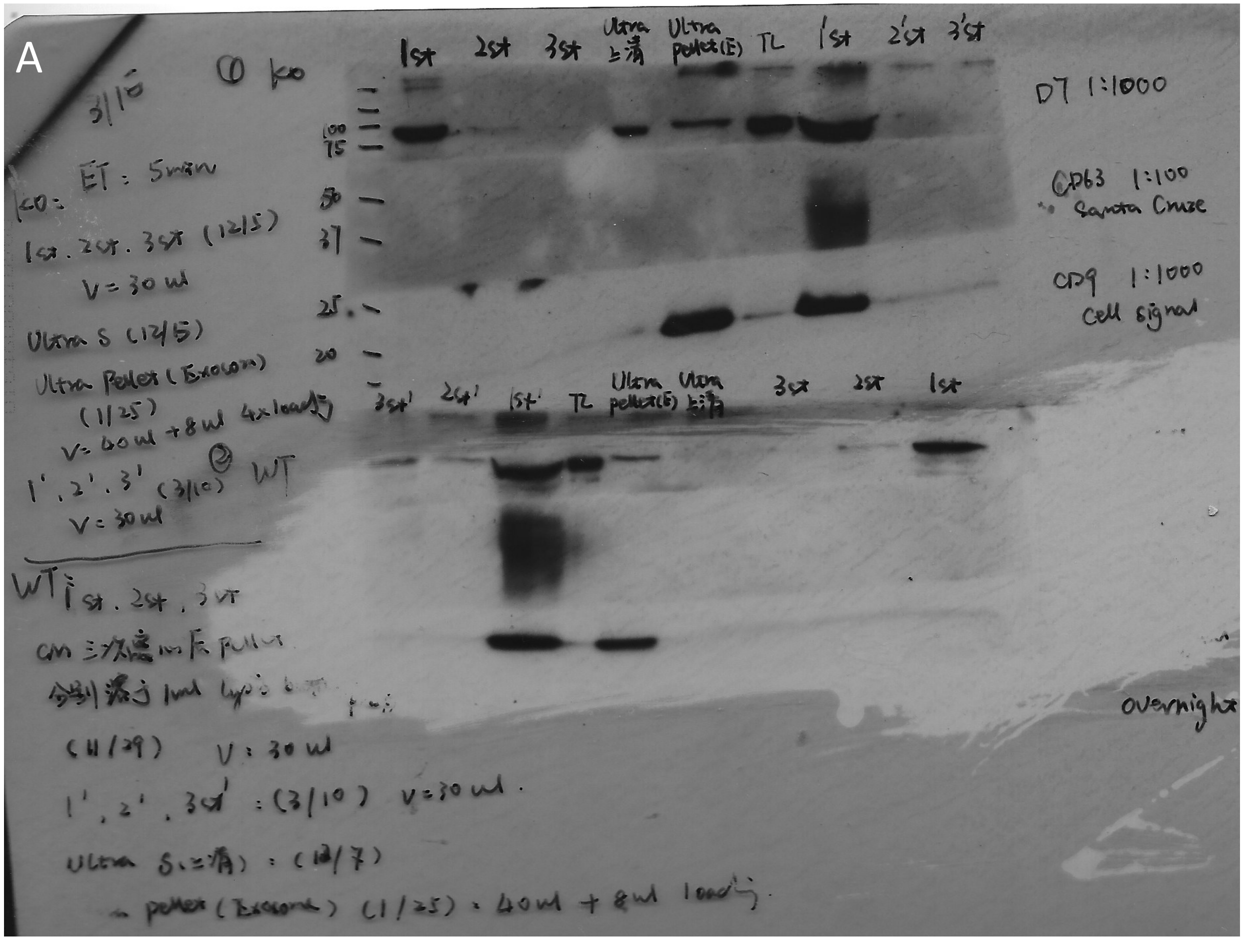

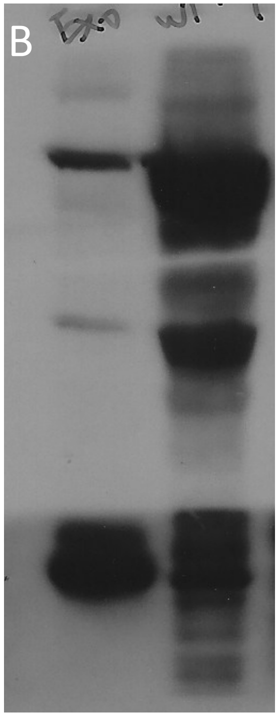

Original films for Figure 2

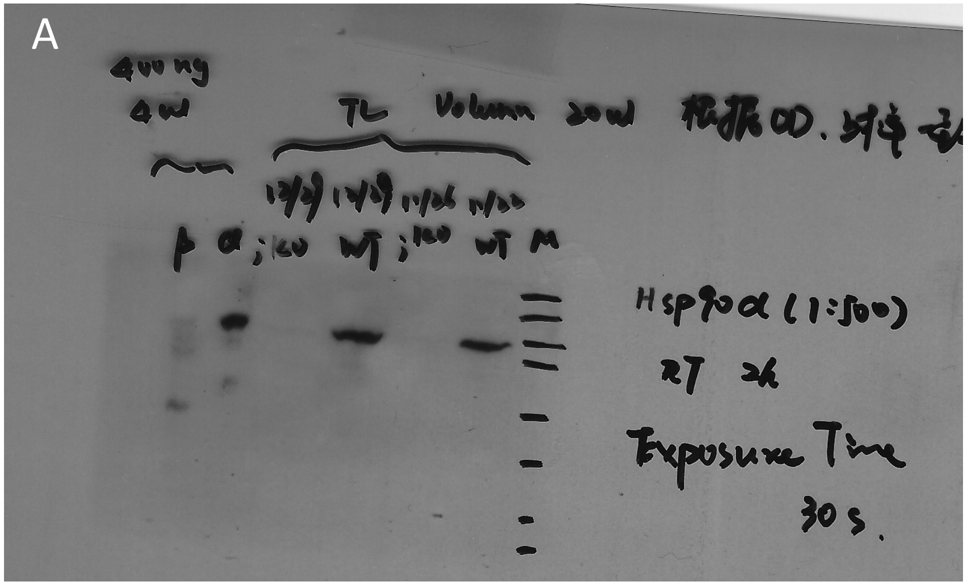

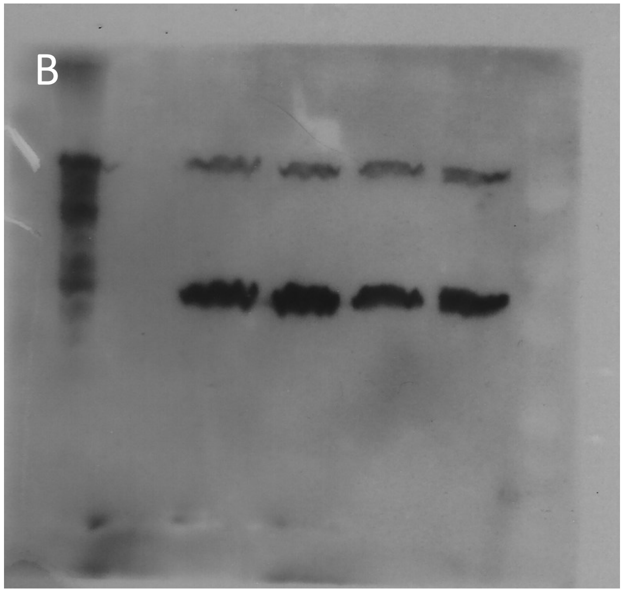

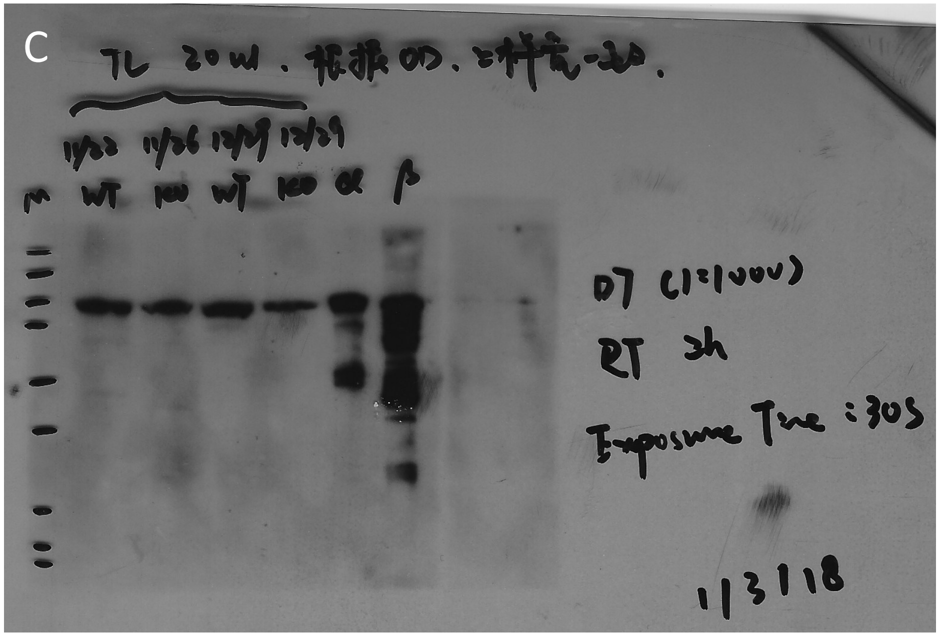

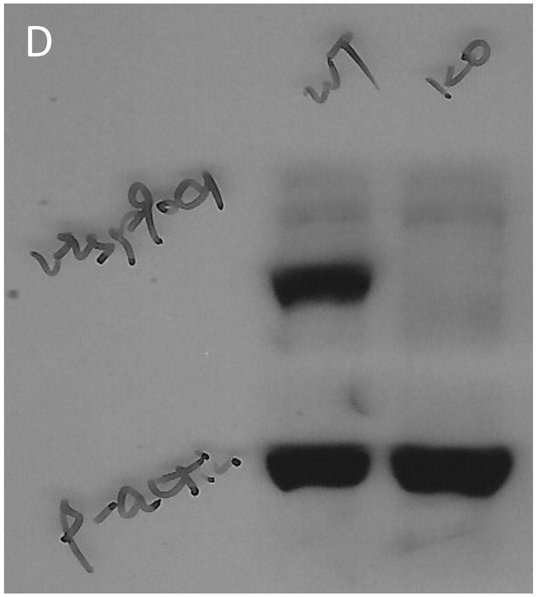

Original films for Figure 2 (continued)

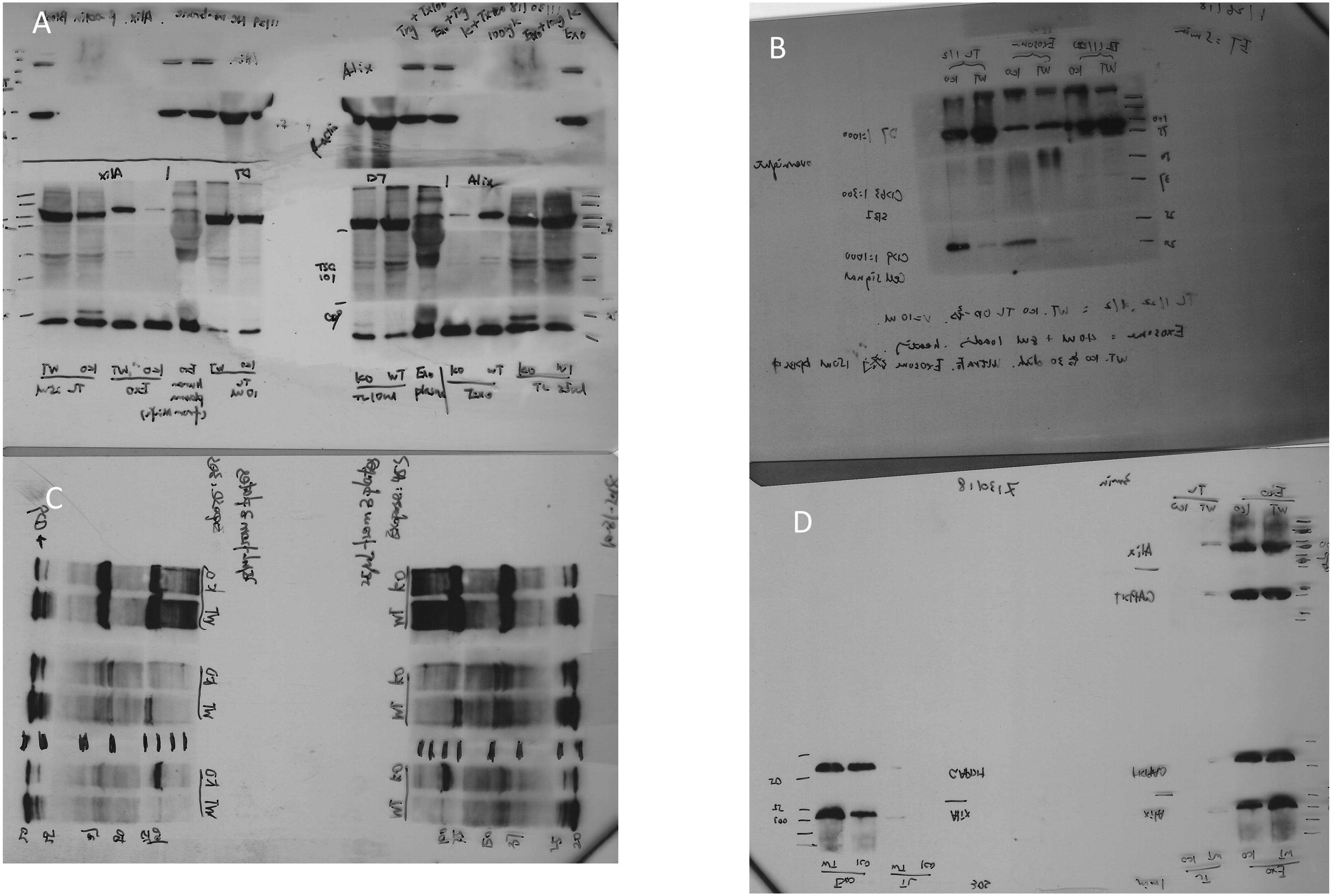

Original films for Figure 4

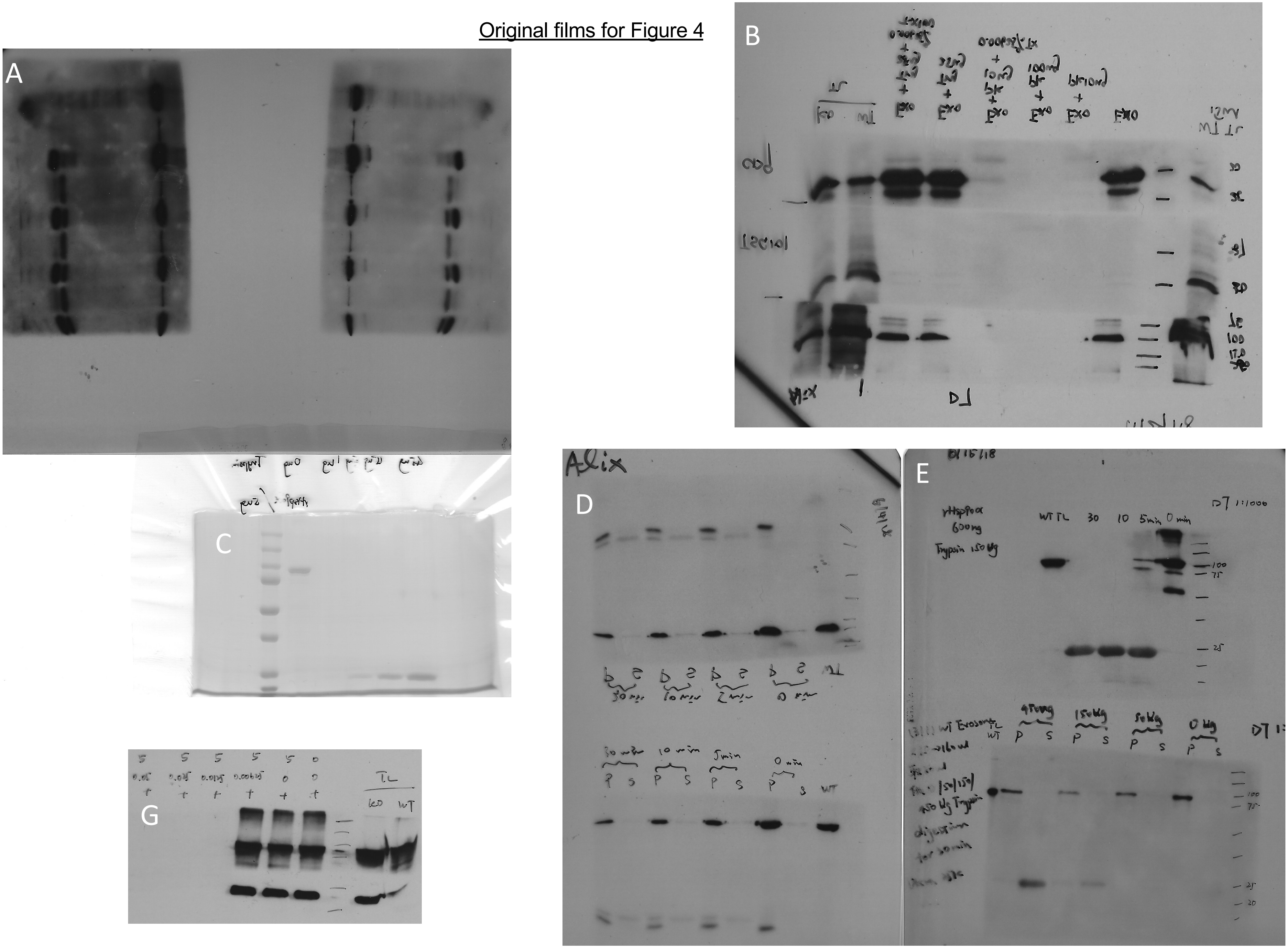

Original films for Figure 5

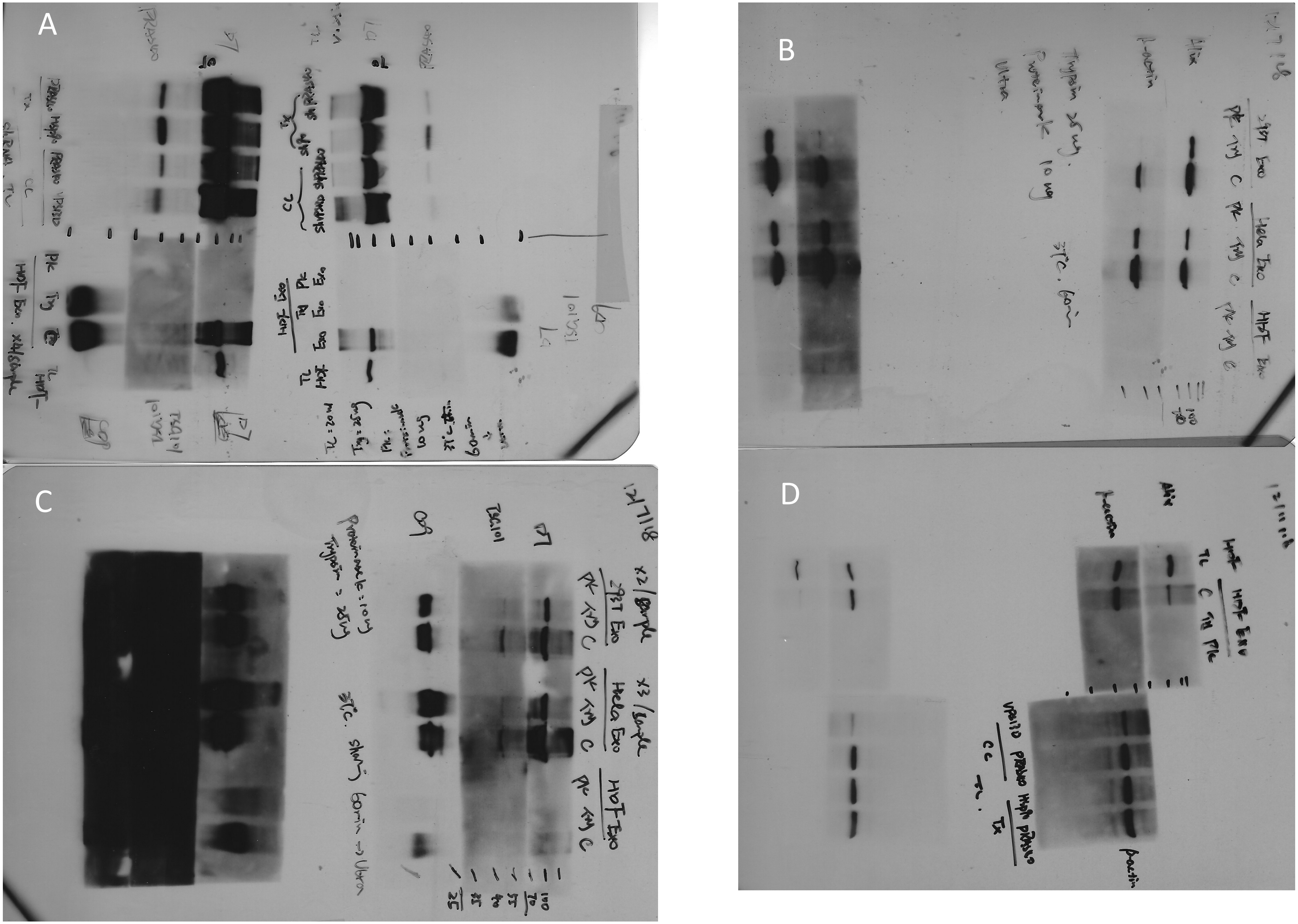

Supplement: Supplementary file 1 — raw data set [file 41598_2019_51704_MOESM1_ESM.pdf]
